# Microstructure and Electrical Properties of Fluorene Polyester Based Nanocomposite Dielectrics

**DOI:** 10.3390/polym13183053

**Published:** 2021-09-10

**Authors:** Wenchao Zhang, Kuo Zhao, Feng Guan, Jinghua Yin, Yu Feng, Jialong Li, Yanpeng Li

**Affiliations:** 1 Key Laboratory of Engineering Dielectrics and Its Application, Ministry of Education, Harbin University of Science and Technology, Harbin 150080, China; wenchao2482206@163.com (W.Z.); iridescen7@163.com (K.Z.); 2 School of Electrical and Electronic Engineering, Harbin University of Science and Technology, Harbin 150080, China; 3 School of Computer Science and Technology, Harbin University of Science and Technology, Harbin 150080, China; guanfeng0130@163.com; 4 School of Material Science and Engineering, Shanxi University of Science and Technology, Xi’an 710021, China; 5 School of Materials Science and Engineering, Harbin University of Science and Technology, Harbin 150080, China; lypharbin@163.com

**Keywords:** dielectric, fluorene polyester, composite film, corona aging lifetime

## Abstract

As a new type of dielectric material, the low dielectric constant and corona resistance life of fluorene polyester (FPE) restricts the range of its applications. In order to simultaneously achieve a high dielectric constant and the long corona aging lifetime of FPE, SiO_2_ nanoparticles were chosen as additive to prepare FPE-based composite films. The microstructure of the composite film was characterized by scanning electron microscopy (SEM), X-ray diffraction (XRD), infrared spectroscopy (IR) and small-angle X-ray scattering (SAXS). The dielectric performances of the composites, including the dielectric constant, breakdown strength and corona resistance lifetime, were investigated. The results show that the introduced SiO_2_ does not destroy the structure of the FPE molecular chain and that it increases the thickness of the filler-matrix interface. The dielectric constant of SiO_2_/FPE composites increased from 3.54 to 7.30 at 1 Hz. Importantly, the corona resistance lifetime increased by about 12 times compared with the pure FPE matrix. In brief, this work shows what possibilities there might be when considering the potential applications of high-strength insulating materials.

## 1. Introduction

Polymers have been widely used in electrical insulation [1,2,3,4], aerospace [5,6,7,8], energy storage [9,10,11] and other fields on account of their excellent electrical properties [12,13,14]. Limited by the low dielectric constant of pristine polymer and the poor corona resistance life, the application of polymers in some insulation or high-*k* fields is, however, restricted [15,16,17]. For ferroelectric polymers, such as PVDF(Polyvinylidene fluoride) and their copolymers, despite the fact that they generally have a relatively high dielectric constant and breakdown strength, their high dielectric loss and poor thermal stability present challenges. Therefore, constructing composite materials with high stability and high dielectric properties has become an important focus of current research. To maintain stability under high temperature and other conditions, polymers containing strong intramolecular or intermolecular forces—bonds such as hydrogen bonds (–NH–, –CO–, –NH–, etc.), conjugated π bonds (imide ring, imidazole ring, etc.), aromatic or heterocyclic aromatic molecular skeletons and high-strength chemical bonds (C–F etc.) [18,19]—seem to be an excellent choice. According to their different chemical structures, dielectric polymers with high thermal stability can be roughly divided into three categories: aromatic polymers, heteroaromatic polymers, and fluorine-containing compounds. Among them, the heteroaromatic polymer is a kind of polymer material that contains both a benzene ring and a heterocyclic structure in the molecular chain [20]. The interaction between the benzene ring and the heterocyclic ring means that this type of dielectric polymer usually has high thermal stability. FPE is a new type of dielectric material, which can be used for high-temperature energy storage. Compared with the most widely used polyimide, which is another heteroaromatic polymer, FPE has a higher dielectric constant (3.5) and a higher breakdown field strength (524 kV/mm). The dielectric loss can be kept less than 0.003 in the range of 25–250 °C. Li et al. [21] prepared FPE films, firstly by using a solution blending method. The prepared film capacitors have higher charge and discharge efficiency; the dielectric properties are less affected by temperature; and the capacitance temperature coefficient (TTC), in the range of 25–250 °C, can be kept less than 0.003. It is significant that, given these advantages of FPE, the dielectric constant of FPE can be further improved without sacrificing its insulation properties.

Introducing nano-scaled inorganic fillers with proper dielectric properties into the polymer matrix is a general method to construct polymer composites with excellent performances. In this topic, the interface between the matrix and the filler is a key factor in improving the performance of the composite film. Lewis pointed out that the interaction region is the conductive layer [22], Nelson constructed the dielectric double-layer structure model [23], and Tanaka further constructed the interface multi-core model theory [24,25,26]. These theories are a source of great inspiration to researchers looking to investigate the interface between the organic phase and inorganic phase. Roy and Nelson [27] prepared SiO_2_/XLPE composite films and found that the composite film can significantly improve the dielectric properties and the dendritic aging properties. Cao and Irwin [28] investigated the dielectric constant and dielectric loss of SiO_2_/polyimide composite film. Their results indicated that the conductivity characteristics of the composite film are consistent with the space charge mechanism. Zhang [29] prepared SiO_2_/PI composite films by the sol-gel method and the solution blending method. The dielectric constant and dielectric loss of the composite films increase with the increase in inorganic content, and the dielectric properties of the films prepared by solution blending have greater stability. These also prove that SiO_2_ is a kind of inorganic particle that can stably and effectively improve the dielectric properties of composite films. In addition, Zha [30] established a corona aging resistance model by studying the effect of nanoparticles on the electrical properties of polymers. They found that the dielectric constant and dielectric loss of the composite film increased with the increase in inorganic particle content, and the corona resistance performance was improved. This was because the introduction of inorganic particles increased the interface area of the composite film, resulting in the enhancement of interface polarization. Gu et al. [31] prepared micron boron nitride/polyamide acid (mBN/PAA) composite film by in situ polymerization, then prepared mBN/PAA electrospun fibers using electrospinning technology, and finally prepared dielectric thermal conductivity mBN/polyimide (mBN/PI) composite film by hot pressing. The composite film thus obtained had relatively high thermal conductivity (0.696 W/mK) and an excellent dielectric constant (3.77). Bian et al. [32] introduced Al_2_O_3_ and BN nanoparticles into epoxy resin, which increased the breakdown time of composite films by 406% compared with pure epoxy resin.

In this work, SiO_2_ nanoparticles were used as additives to prepare an FPE-based composite film. The microstructure of the composite film was characterized by SEM, XRD, IR and SAXS. The dielectric properties of the composite film, including its dielectric constant, breakdown strength and corona life, were investigated. The results indicate that the dielectric constant and the corona resistance life of the composites have been significantly improved. In brief, the FPE-based composite film with high dielectric properties may make a significant contribution in prospective applications of electrical insulation materials.

## 2. Materials and Methods

### 2.1. Materials

The diameter of the SiO_2_ nanoparticles used in this experiment was 30 nm. They were purchased from the Aladdin Company (Shanghai, China), while the NMP (*N*-methylpyrrolidone) solvent was purchased from Tianjin Fuyu Fine Chemical Co., Ltd. (Tianjin, China), and the FPE particles were purchased from PolyK (Philipsburg, PA, USA). The above experimental articles were used directly in the experiment without further purification.

### 2.2. Preparations

**Figure 1:**
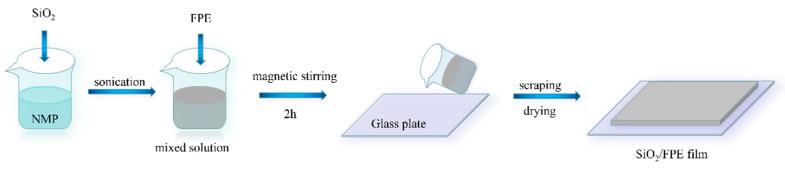
Schematic diagram of SiO_2_/FPE composite solution-blending preparation method.

Pure FPE and SiO_2_/FPE composite films with mass fractions of 3, 5, 7 and 9 wt% were prepared using the solution blending method, as shown in Figure 1. According to the different mass fractions, silica particles were weighed and placed in 3.5 mL NMP solution. Firstly, through ultrasonic treatment for 1 h, the particles were fully dispersed. Then, 0.4 g FPE particles were added to the NMP solution, and this was then stirred for 2 h with a magnetic mixer. The glass plate was coated with the solution and the film was scraped with a film wiper. The plate was then dried in a vacuum oven at 80 °C for 12 h and then at 120 °C for 12 h. Finally, an FPE-based composite film with a thickness of 20 μm was obtained.

### 2.3. Characterizations

In this work, the microstructures of SiO_2_ nanoparticles and SiO_2_/FPE composite films were observed under vacuum mode by a 20 kV SEM produced by the Philips company in the Netherlands (Amsterdam). Phase properties and crystallinity of the composite film were tested using a Bruker D8 wide-angle XRD instrument. The changes in functional groups on the composite films were tested by Fourier transform infrared spectroscopy(FTIR). SAXS experiments were carried out on the beam line 1W2A of the Beijing Synchrotron radiation device to explore the thickness and fractal dimension of the composite film interface layer. The storage ring operated at 2.5 GeV with a current of about 80 mA. In order to monitor the structural changes during nonaxial deformation, the charge-coupled device Mar165-CCD was used to collect 2D scattering images in real time. The distance from the sample to the detector in the beam direction was 1500 mm. The dielectric properties of the composite films were measured by a new controlled broadband dielectric spectrometer (concept 40). The measurable frequency range was 2 μHz–20 MHz, the impedance range 0.01 Ω–100 TΩ, the capacitance range 1 Ff–1 F, the phase difference accuracy 2 × 10^−3^ and the loss accuracy Tan(δ) < 10^−5^. In this study, the test frequency range was 10^0^ to 10^7^ Hz. According to IEC-60343, the corona aging resistance of the composite film was tested in an electric field of 60 kV/mm, and the thickness of the SiO_2_/FPE composite film was measured by a digital thickness gauge with an accuracy of 0.001 mm. By vacuum evaporation, aluminum electrodes (25 mm in diameter) were deposited on both sides of the sample for subsequent dielectric measurements. The AC (Alternating Current) breakdown strength test of the nanocomposite material was carried out using the IEC 243 equipment.

## 3. Results and Discussion

**Figure 2:**
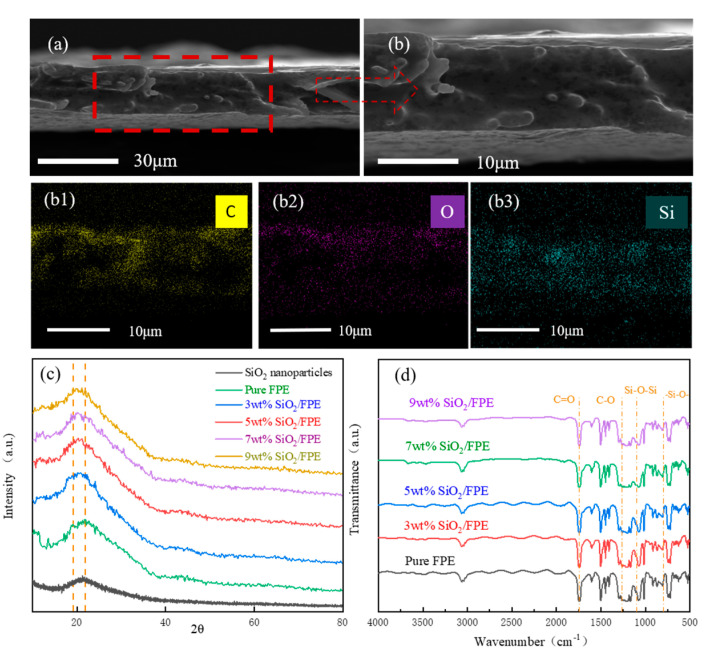
SEM images of a fractured cross-section of the composite film are shown in (**a**,**b**); elements mapping of SiO_2_/FPE composite film in (**b1**–**b3**); XRD images of SiO_2_ nanoparticles and SiO_2_/FPE in (**c**); and FTIR images of SiO_2_/FPE in (**d**).

The SEM images of a fractured cross-section of the composite film at 9 wt% are shown in Figure 2 (Figure 2b is a magnification of Figure 2a). The thickness of the composite film is approximately 20 μm, which suggests that the flatness of the composite film is relatively smooth. Figure 2b1–b3 show the element mapping of the composite film and the distribution of C, O and Si. As shown in Figure 2b, similar curves were observed for all samples. Figure 2c shows the XRD pattern of the composite film. It can be clearly seen that there is no special peak in SiO_2_, indicating that it is an amorphous particle. With the addition of SiO_2_, the diffraction peak at about 20° gradually decreases, and the position of the absorption peak shifts to a low angle. This indicates that the nanoparticles were successfully combined. According to the Prague formula, the introduction of SiO_2_ also expanded the FPE molecular chain spacing. Compared with the results of other scholars, the dispersion of particles in the composite film that we prepared is more uniform, while the peak position in the XRD image is close to the same [33,34]. The infrared spectrum of the composite film is shown in Figure 2d. The absorption peak at 1742 cm^−1^ corresponds to the C=O stretching of the ester group, and the absorption peaks at 1262 cm^−1^ correspond to the C–O stretching. It can be found by referring to other research work that the absorption peak appears at 1095 cm^−1^, this being caused by the anti-symmetric stretching vibration of the Si–O–Si bond. For SiO_2_, the peak at 800 cm^−1^ is attributed to –Si–O– [35,36]. This indicates that the characteristic chemical structure of fluorene polyester still exists in SiO_2_/FPE composites and that the introduction of SiO_2_ does not destroy the structure of FPE itself. It also shows that SiO_2_ nanoparticles were successfully introduced into the FPE matrix. This may prove to be an important basis for the excellent electrical properties of composite films. The diameter of SiO_2_ particles is 30 nm, as shown in Figure S1.

**Figure 3:**
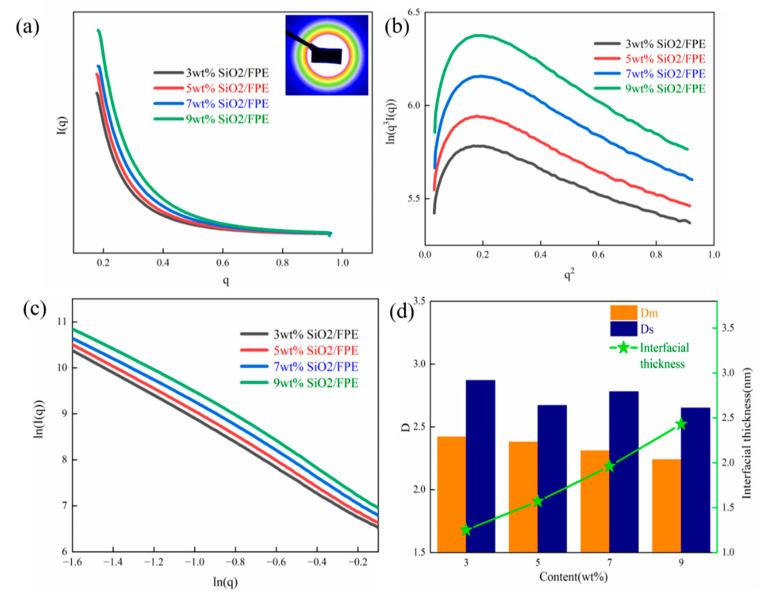
(**a**) The SAXS scattering intensity curves of the SiO_2_/FPE composite film with different concentrations. (**b**) Porod curves of the SiO_2_/FPE composite film. (**c**) Typical ln (I(q)) versus ln (q) plots of the SiO_2_/FPE composite film. (**d**) The fractal dimension and interfacial thickness of the SiO_2_/FPE composite film.

SAXS is considered an effective means to explore the microstructure of polymer-based composite films [37,38]. The purpose of the SAXS test is to investigate the thickness and the fractal dimension of the interface layer between SiO_2_ particles and the FPE matrix. Figure 3a shows the SAXS scattering intensity curve of SiO_2_/FPE composites with different mass fractions. It is obvious that the scattering intensity decreases with the increase in the scattering vector. In the composite films, with the continuous increase in silica components, the electron density in SiO_2_ nanoparticles and the FPE matrix are also increasing, as is the scattering intensity of the composite film. Figure 3b shows the Porod curve obtained based on the classical SAXS theory. The end of the Porod curve shows a negative deviation trend, indicating that an interface layer is formed between the SiO_2_ and FPE molecular chains. In the case of infinite slit alignment, the relationship curve of ln [I (q)] ~ln (q) is as displayed in Figure 3c. As the figure shows, each sample has two linear regions, which proves that there are fractal features in the composite. Generally speaking, the fractal is the research object of self-similarity in the morphology, function and information of composite films. The fractal dimension of a composite film is an important parameter of microstructure, and SAXS is an effective method to measure the fractal dimension. According to Porod’s law, the thickness of the interface between SiO_2_ nanoparticles and the FPE matrix can be calculated [39].

(1) $$\ln[q^{3}I(q)]=\ln k^{\prime }-\delta^{2}q^{2}$$

(2) $$E=\sqrt{2\pi }\sigma$$

where *E* is the average thickness of the interface layer, and σ represents the slope of the curve segment and the absolute value of the ending. According to the above formula, the surface fractal (D_s_) and mass fractal (D_m_) of the composite film and the thickness of the interface layer can be obtained, as shown in Figure 3d. The density of mass distribution is negatively correlated with the D_m_ value, and the surface roughness is positively correlated with the D_s_ value [40,41]. According to the Porod curve and the ln [I (q)]-ln (q) curve, the thickness and fractal dimension of the interface layer are obtained. It can be observed from Figure 3d that the incorporation of SiO_2_ nanoparticles not only increases the thickness of the interface layer but also enlarges the molecular structure of the FPE matrix.

**Figure 4:**
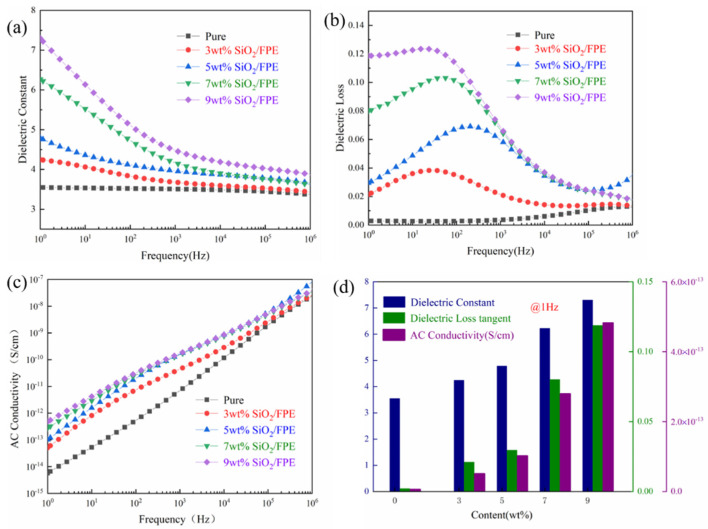
(**a**) Dielectric constant. (**b**) Dielectric loss tangent. (**c**) AC conductivity of pure FPE and SiO_2_/FPE composite films as a function of frequency. (**d**) Dielectric constant, dielectric loss tangent, AC conductivity of composite films at 1 Hz.

Figure 4a shows the relationship between the dielectric constant of pure FPE and the SiO_2_/FPE composite films as a function of frequency. It can be clearly seen that with the increase in the SiO_2_ filler content, the dielectric constant of the composite film gradually increases. First, according to the results of SAXS, the introduction of SiO_2_ nanoparticles loosens the structure of the composite film, which improves the responsiveness of the composite film to the electric field. At the same time, the dielectric constant of the composite film is also affected by the Maxwell-Wagner-Sillars (MWS) effect [42] and space charge. With the continuous introduction of SiO_2_ nanoparticles, more interfaces and space charges are introduced inside the composite film, which also increases the possibility of raising the dielectric constant. As shown in Figure 4d, when the filler content is 9 wt%, the dielectric constant of the composite material is 7.30 at 1 Hz, which is nearly 200% higher than that of FPE.

As shown in Figure 4b, the dielectric loss of the composite film maintains a strong dependence on frequency. The dielectric loss (*ε*″) is a physical quantity used to describe the energy loss in dielectric materials. The energy loss mainly comes from the following three aspects: conductivity loss (transmission type loss), slow polarization loss (dipole loss) and interface polarization loss. For composite films, *ε*″ can be expressed as the following formula [43,44,45]:

(3) $$\varepsilon^{\prime \prime }={\varepsilon_{dc}}^{\prime \prime }+{\varepsilon_{MW}}^{\prime \prime }+{\varepsilon_{D}}^{\prime \prime }$$

where *ε_d_*″ and *ε_MW_*″ represent the conductance loss and interface polarization loss, respectively; *ε_D_*″ represents the dipole loss; and the expression of the conductance loss is:

(4) $${\varepsilon_{dc}}^{\prime \prime }=\frac{\sigma_{dc}}{2\pi f}$$

where *σ_dc_* and *f* represent direct current conductivity and frequency, respectively. According to Formula (4), after taking the logarithm of *ε_dc_*″ and *f* at the same time, *ε_dc_*″ will become a straight line. The expression for interface polarization is:

(5) $${\varepsilon_{MW}}^{\prime \prime }\propto \left(1+\frac{K}{1+{\left(2\pi f\right)}^{2}\tau^{2}}\right)$$

*K* represents the dielectric constant of the composite material at the interface, and τ represents the relaxation time of the interface polarization. According to Formula (5), by taking the logarithm of both sides of the equation, it can be found that *ε_MW_*″ presents an inverse curve function. It is worth noting in Figure 4b that in the low frequency range of 10^2^ Hz, as the content of additives continues to increase, the loss tangent of the composite film becomes closer to a straight line. Usually, the high-frequency dielectric loss of dielectric mainly comes from the polarization of the dipole, and the dielectric loss comes from the direct current conductance loss and the interface polarization in the low frequency. Based on the above theory, it can be concluded that with the increase in the composition, the linear characteristic of the curve of dielectric loss of the composite film becomes more obvious, which proves that the DC conduction loss inside the composite film becomes more dominant as the filler content increases. At the range of 10^3^–10^4^ Hz, the rotation of the dipole cannot keep up with the change in frequency, and the dielectric tangent loss curve at this stage shows a downward trend. Above 10^5^ Hz, the dielectric loss tangent tends to stabilize due to the polarization of the dipole orientation. It can be seen from Figure 4d that when the SiO_2_ content reaches 9 wt%, the loss tangent of the composite material reaches the maximum value of 0.12, but this value remains at a relatively low level.

Figure 4c shows the relationship between AC conductivity and frequency. AC conductivity also increases with frequency and filler content. Due to the introduction of a large number of SiO_2_ nanoparticles, charge carriers can be generated between the interface layers of the composite material. Due to the bridging effect between the nanoparticles, the distance of the charge transition is also shortened, so the AC conductivity increases. When the frequency is 1 Hz, the maximum AC conductivity of the composite is still in the range of 10^−12^ S/cm, indicating that the insulation of the composite is still excellent.

The breakdown field strength is also an important factor to measure the insulation performance of a composite film. The characteristic breakdown field strength of each sample was calculated using the two-parameter Weibull distribution function. The calculation formula is as follows:

(6) $$P=1-\exp\left[-{\left(\frac{E_{b}}{E_{0}}\right)}^{\beta }\right]$$

**Figure 5:**
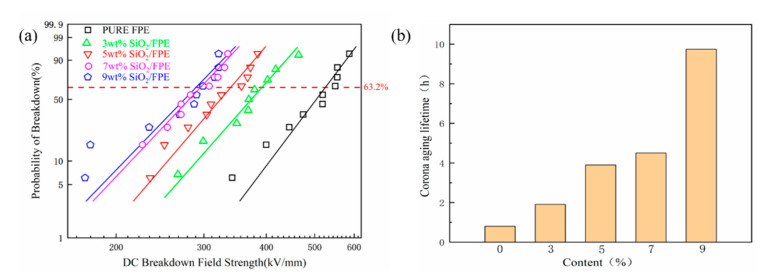
(**a**) Breakdown strength of the SiO_2_/FPE composite films. (**b**) The corona aging lifetime of pure FPE and SiO_2_/FPE films with different concentrations of SiO_2_.

*P* represents the cumulative probability of electrical failure, *E*_b_ represents the breakdown strength, *E*_0_ is the breakdown strength with a cumulative failure probability of 63.2%, and *β* is the shape parameter related to data scattering. Figure 5a shows the breakdown strength of the SiO_2_/FPE composite films. It can be clearly seen that the breakdown field strength of the composite film decreases with the increase in the content of SiO_2_. According to the results of SAXS, it is obvious that the introduction of SiO_2_ nanoparticles expanded the molecular spacing of the FPE matrix. The microstructure of the composite film is looser, and the free volume in the matrix becomes larger. This may be the main reason why the breakdown field strength decreases rapidly. Meanwhile, the introduction of the SiO_2_ nanoparticles may also form some physical changes at the interface. The mismatch of the dielectric constant between the SiO_2_ nanoparticles and the FPE matrix will also cause electric field distortion. All of these factors lead to the decrease in breakdown field strength in the composite films. It is also important to measure the mechanical properties of composite films. The existence of physical defects leads to a decline in the mechanical properties of the composite films, similar to the breakdown properties. The tensile performance of composite films is shown in Figure S2. The pure FPE has an excellent breakdown field strength of 524 kV/mm. When the filler content is 9 wt%, the breakdown field strength is 288 kV/mm, which is still outstanding in common polymers.

The corona aging lifetime of the composite under the electric field strength of 60 kV/mm is shown in Figure 5b. With the increasing filler content of SiO_2_ nanoparticles, the corona aging lifetime also has an obvious upward trend. The essence of the corona aging test is the process of electrical erosion on composite films. According to the SEM and EDS images of composite films, it is obvious that the SiO_2_ nanoparticles are uniformly distributed in the FPE matrix. When the FPE matrix is eroded, SiO_2_ nanoparticles are exposed and form an inorganic protective layer. This is the reason why the corona aging lifetime decreases with the filler content of SiO_2_ nanoparticles. With the change in mass fraction from 0 wt% to 9 wt%, the corona resistance time is increased from 0.8 h to 9.75 h, and the corona aging lifetime of the composite is about 12 times higher than that of the pure FPE film. In addition, the FTIR image of the composite film after the corona test is displayed in Figure S3.

**Figure 6:**
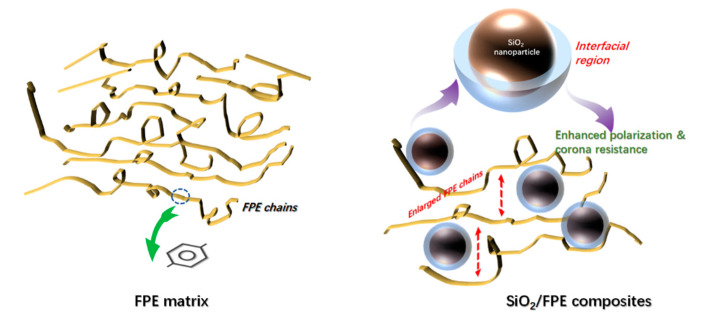
Schematic diagram of the role of SiO_2_ particles in an SiO_2_/FPE composite film.

As shown in Figure 6, after introducing SiO_2_ nanoparticles into FPE, it can be seen from the XRD and SAXS results that the molecular chain in the FPE matrix is enlarged and that the internal structure of the composite film becomes loose, which makes the free volume in the composite film larger and leads to a slight decrease in the breakdown performance of the composite film. It can also be found from the SAXS results that there is an interface layer between the inorganic filler and the polymer matrix, which enhances the interfacial polarization strength of the composite film. This is key to improving the dielectric properties and long-term breakdown performance of composite films.

## 4. Conclusions

In this study, SiO_2_ particles were dropped into an FPE matrix using the solution blending method to improve the dielectric and insulation properties of the composite dielectric. The SAXS test shows that the incorporation of SiO_2_ particles makes the structure of the composite film looser and increases the interface thickness between the FPE matrix and SiO_2_ particles. The dielectric constant of the SiO_2_/FPE composite film also increases with the increase in silica content. When the mass fraction of SiO_2_ is 9 wt%, the dielectric constant reaches 7.3, and the dielectric loss and conductivity remain within the acceptable range of engineering application. At the same time, the composite film still maintains excellent corona resistance. Therefore, not only do SiO_2_/FPE composite films have a simple preparation process, but they also have excellent dielectric and insulation properties, which may be of great help in the preparation of electrical insulation and flexible semiconductor devices in the future.

## Data Availability

Not applicable.

## Supplementary Materials

The following are available online at https://www.mdpi.com/article/10.3390/polym13183053/s1, Figure S1: The SEM image of SiO_2_ nanoparticles, Figure S2: The stress-strain curve of the SiO_2_/FPE composites, Figure S3: The FTIR of composite films before and after corona test.
