# Supplementary material for: Microstructure and Electrical Properties of Fluorene Polyester Based Nanocomposite Dielectrics
Source: Polymers (Basel). 2021 Sep 10;13(18):3053. doi: 10.3390/polym13183053 (PMC8471433; doi:10.3390/polym13183053)
Supplement: Supplementary file 1 [file polymers-13-03053-s001.zip › polymers-1357429-supplementary.pdf]

Supplementary Materials

Microstructure and Electrical Properties of Fluorene Polyester Based Nanocomposite Dielectrics

Wenchao Zhang [1,2], Kuo Zhao [1,2], Feng Guan [3], Jinghua Yin [1,2,*], Yu Feng [1,2,*], Jialong Li [4,*] and Yanpeng Li [5]

[1]Key Laboratory of Engineering Dielectrics and Its Application, Ministry of Education, Harbin University of Science and Technology, Harbin 150080, China; wenchao2482206@163.com (W.Z.); iridescen7@163.com (K.Z.);

[2]School of Electrical and Electronic Engineering, Harbin University of Science and Technology, Harbin 150080, China;

[3]School of Computer Science and Technology, Harbin University of Science and Technology, Harbin 150080, China; guanfeng0130@163.com (F.G.)

[4]School of Material Science and Engineering, Shaanxi University of Science and Technology, Xi'an 710021, China;

[5]School of Materials Science and Engineering, Harbin University of Science and Technology, Harbin 150080, China; lypharbin@163.com (Y.L.)

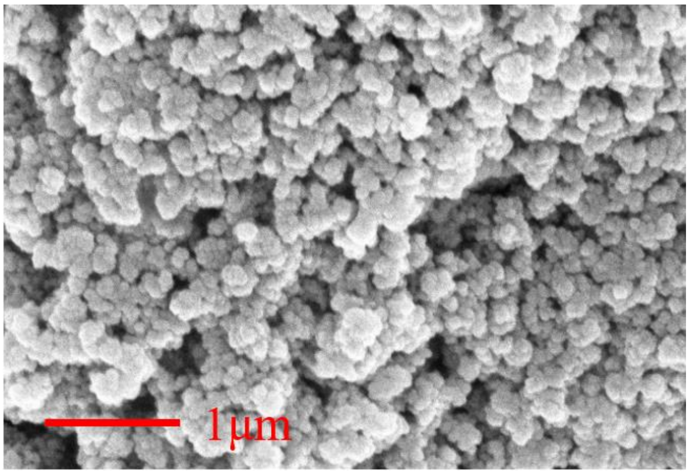

Figure S1 The SEM image of SiO_2 nanoparticles

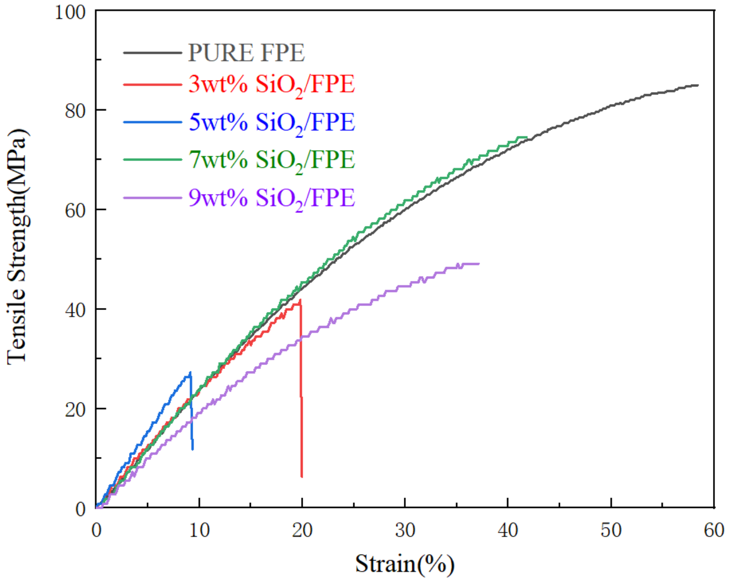

Figure S2 The stress-strain curve of the SiO_2/FPE composites

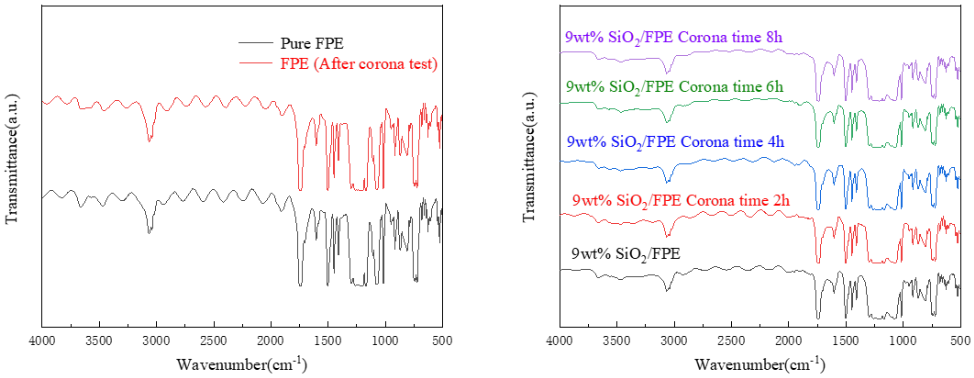

Figure S3 The FTIR of composite films before and after corona test
